# Asporin Reduces Adult Aortic Valve Interstitial Cell Mineralization Induced by Osteogenic Media and Wnt Signaling Manipulation *In Vitro*

**DOI:** 10.1155/2020/2045969

**Published:** 2020-04-10

**Authors:** Anisha Polley, Riffat Khanam, Arunima Sengupta, Santanu Chakraborty

**Affiliations:** ^1^Department of Life Sciences, Presidency University, Kolkata 700073, India; ^2^The Department of Life Sciences and Biotechnology, Jadavpur University, Kolkata 700032, India

## Abstract

Worldwide, calcific aortic valve disease is one of the leading causes of morbidity and mortality among patients with cardiac abnormalities. Aortic valve mineralization and calcification are the key events of adult calcific aortic valve disease manifestation and functional insufficiency. Due to heavy mineralization and calcification, adult aortic valvular cusps show disorganized and dispersed stratification concomitant with deposition of calcific nodules with severely compromised adult valve function. Interestingly, shared gene regulatory pathways are identified between bone-forming cells and heart valve cells during development. *Asporin*, a small leucine-rich proteoglycan (43 kDa), acts to inhibit mineralization in periodontal ligament cells and is also detected in normal murine adult aortic valve leaflets with unknown function. Therefore, to understand the Asporin function in aortic cusp mineralization and calcification, adult avian aortic valvular interstitial cell culture system is established and osteogenesis has been induced in these cells successfully. Upon induction of osteogenesis, reduced expression of *Asporin* mRNA and increased expression of bone and osteogenesis markers are detected compared to cells maintained without osteogenic induction. Importantly, treatment with human recombinant Asporin protein reduces the mineralization level in osteogenic media-induced aortic valvular interstitial cells with the concomitant decreased level of Wnt/*β*-catenin signaling. Overall, all these data are highly indicative that *Asporin* might be a novel biomolecular target to treat patients of calcific aortic valve disease over current cusp replacement surgery.

## 1. Introduction

Cardiac diseases are often associated with structural and functional insufficiency of adult aortic valvular cusps [1, 2]. These are mostly characterized by aortic valve mineralization, followed by calcification and subsequent dysfunction with severe morbidity and mortality in humans [3, 4]. According to World Health Organization, it is estimated that 17.9 million people (31%) died from different cardiovascular diseases in the year 2017. Prevalence of calcific aortic valve disease (CAVD) accounts for around 5-25% of all types of cardiovascular diseases. A large number of research efforts are directed towards better understanding of the CAVD mechanism. CAVD is projecting around 13% of global population and persisting as a serious health concern, worldwide. Approximately 25% of people over age of 65 have increased risk of CAVD. Human CAVD is characterized by the accumulation of calcium salts on aortic valvular cusps due to genetic and various environmental factors with severely compromised valvular function [5–7].

During valvulogenesis, embryonic endocardial endothelial cells of outflow tract (OFT) cushions get differentiated to form mature, stratified, tri-layered aortic valvular cusp in adult hearts [8]. During this developmental event, highly proliferative, migratory, and undifferentiated cells of endocardial cushion give rise to mature aortic valves with stratified extracellular matrix (ECM) [9, 10]. Mature valve leaflets are stratified into elastin-rich atrialis/ventricularis, proteoglycan-rich spongiosa, and highly organized collagen fiber-rich fibrosa [5]. CAVD is mainly associated with complete disarray and disorganization of this trilaminar structure and ECM components in fibrosa. It has been reported that expression of genes associated with bone and cartilage development is also elevated in patients with CAVD condition [11]. Previous reports have suggested the existence of shared gene regulatory pathways between developing heart valve and bone-forming cells [12]. Valvular cells are of two heterogeneous types, namely valvular endothelial cells (VECs) and valvular interstitial cells (VICs) [13]. These VICs have the potential to differentiate into preosteoblasts upon several pathological cues with relatively unknown mechanism.

Asporin, also known as periodontal ligament-associated protein1 (PLAP1), is a member of small leucine-rich proteoglycan (SLRP) family of ECM proteins [14, 15]. Previously, it is reported that *Asporin* is expressed in mouse and human tissues including the osteoarthritic articular cartilage, aorta, uterus, heart, and liver [12, 15–17]. In addition, recent reports demonstrated strong expression of *Asporin* mRNA in adult mouse aortic valve leaflets, although without any functional relevance [12]. It plays an important role in the negative regulation of bone cell calcification process via inhibition of bone morphogenetic protein 2 (BMP2)-Smad/1/5/8 signaling pathway via competitive binding with Bmp receptor and results in reduced level of calcification in periodontal cells [16]. Wnt/*β*-catenin signaling has multiple and diverse roles in embryonic development including heart and bone formation. Importantly, it is reported that expressions of Wnt/*β*-catenin pathway-specific genes are increased during CAVD and promotes osteogenesis [18]. Asporin-dependent regulation of aortic valve mineralization with a concomitant expression of procalcific Wnt/*β*-catenin signaling has not been well studied before, especially in the case of adult calcific aortic valve disease or CAVD. Therefore, studies on the regulatory mechanism between *Asporin* and Wnt/*β*-catenin signaling in the valvular mineralization process might be worth exploring in the purview of CAVD therapeutics.

Here, in this report for the first time, we have demonstrated the Asporin-mediated inhibition of mineralization level in cultured adult avian aortic valvular interstitial cells (AVICs) induced with osteogenesis. In addition, our data are also indicative of Asporin functions upstream of mineralization and calcification promoting pathways active in cultured AVICs with induced osteogenesis.

## 2. Materials and Methods

### 2.1. Avian Heart Collection

Fertilized chick embryos at indicated embryonic time points were obtained from a local government registered hatchery (State Poultry Farm, Tollygunge, Kolkata, West Bengal), and adult chicken hearts were procured from a local slaughterhouse. The specific avian breed used was *Gallus gallus domesticus* (Rhode Island Red and Black Australorp) [19]. Importantly, no animal ethics/usage approvals were needed as no animals were maintained for breeding purpose at the Presidency University. Although procurement of all fertilized eggs and adult hearts was performed in accordance with Presidency University purchase guidelines.

### 2.2. RNA Isolation and Reverse-Transcriptase PCR

Total RNA was isolated from dissected and pooled tissues which are 10-12 embryonic day 5.0 (E5) OFT cushion tissues, adult ventricular tissue, 5-6 adult aortic cusps, in every case of the avian experimental group using 200 *μ*l Trizol (Invitrogen, 15596026). Then 2 *μ*g of total RNA was reverse transcribed for cDNA synthesis by Bio-Rad kit (iScript™ Reverse Transcription Supermix for RT, 170-884) in 20 *μ*l of total volume according to manufacturer's supplied protocol. Total RNA was also isolated from valvular interstitial cell (VIC) cultures using 200 *μ*l Trizol. Then cDNA was synthesized, taking 1 *μ*g of RNA by Reverse Transcription Supermix. RT-PCR was performed in 20 *μ*l of total volume using DNA Taq polymerase (Bioline, BIOTAQ DNA polymerase BIO-21040). These PCR were performed for 35 cycles using 20 pmol of the following primer pairs (IDT) mentioned in Table 1. For each primer set standardization and optimization and to detect the optimum annealing temperature, gradient PCR reactions were done as shown in supplementary figure I.

### 2.3. Quantitative Real-Time PCR (qRT-PCR) Analyses

Quantitative real-time PCR (qRT-PCR) was used for quantitative assay to validate gene expression changes. Here, we have also demonstrated primer efficiencies of three essential gene primers (*Asporin*, *β-catenin*, and *Fibronectin*), used extensively in our data analyses. We have followed the Qiagen guidelines to derive “efficiency and slope” of the primer pairs against cDNA targets [20]. For cDNA synthesis, we have taken 1 *μ*g of template RNA. Then cDNA was serially diluted with nuclease free water as follows: 1 : 1, 1 : 10, 1 : 100, 1 : 1000, and 1 : 10000. Then, standard curves were plotted taking the log of cDNA dilution and Cq values (supplementary figure II). From the standard curve, slope (*S*) was calculated and efficiency was determined by the formula: Primer efficiency (%) = [10∧(−1/S) − 1]^×^100.

Quantitative real-time PCR was performed in 10 *μ*l of total volume using the Bio-Rad real-time PCR kit (SSO fast Eva green super mix, 172-52 03AP) according to previously standardized protocol [19]. Gene expression levels were determined by a Cq value obtained from the Bio-Rad CFX manager software normalized to *β-actin* expression. Each qRT-PCR result represents both biological and technical triplicates (*n* = 3). For avian valvular interstitial cells (AVICs) in culture, gene expression was analyzed as increased or decreased fold change calculated from experimental Cq values generating *∆*Cq and *∆∆*Cq. Statistical significance was calculated for observed differences by Student's *t*-test and considered significant for *p* ≤ 0.05 values.

### 2.4. Adult Chicken Aortic Valvular Interstitial Cell (AVIC) Culture and Osteogenic Induction

Primary aortic valve interstitial cells (AVICs) were established from aortic valvular cusps of adult chicken hearts. These cells were isolated following standardized protocol and cultured on collagen coated plates and chamber slides as described previously by others [9]. Briefly, adult valvular leaflets from the aortic base region were located and dissected away using fine forceps and scissors as needed. Later, with the help of a cotton swab, the upper VECs layer was scraped out from the dissected cusps. Next, the cusps were explanted and subjected to treatment with collagenase II (Sigma 234153) containing M199 medium (Invitrogen, 11150-059) supplemented with 1% penicillin/streptomycin (Pen-Strep) cocktail (Invitrogen, 15140122) at 37°C, 4 × 7 mins in a shaking water bath [9]. Pooled supernatants from 7-8 valves were centrifuged at 800 rpm for 5 mins and the pellet was resuspended in 3 ml complete M199 culture medium which contains M199 medium +10% Fetal Bovine Serum (FBS) and 1% Pen-Strep. Then the final cell suspension was sieved through nylon mesh (local made) and plated onto 0.01% collagen- (Collagen I rat tail-, 3 mg/ml, A10483-01) coated plates containing complete M199 media followed by 2-5 days of culture. After establishing, AVIC explant cultures without any contaminations were used further to perform all the experiments.

To study osteogenic gene induction, cultured cells were treated with osteogenic media containing complete M199 medium, *β*-glycerophosphate (10 mM), L-ascorbic acid (10 nM), and dexamethasone (0.1 *μ*M) as described before [9]. The osteogenic (OS) induction media was added after 3 days of VICs in culture and kept in treatment for a maximum of 4 days (96 hrs) in healthy condition.

### 2.5. Activator and Inhibitor Assays in Adult Avian Valvular Interstitial Cells (AVICs) in Culture

To supplement the Asporin protein in AVICs in culture, the cells were treated directly with the recombinant human Asporin protein (150 ng/ml; Abcam 132382) for 24 hrs in the presence of OS induction media for 4 days. For canonical Wnt signaling pathway manipulation, cultured cells were treated with the recombinant human Wnt3A (rhWnt3A) protein (150 ng/ml; Abcam ab81484) for 24 hrs to activate Wnt signaling, performing activation assay. Similarly, cultured cells were treated with Xav939, also for 24 hrs (2 *μ*M; Abcam ab120897) for Wnt signaling inhibition assay [19, 21]. After completion of all the treatments, experiments were carried out for either total RNA isolation for quantitative mRNA expression analyses or cell fixation for immunostaining. Also, qualitative and quantitative Alizarin Red staining was carried out to compare mineralization of treated cells with untreated controls (*n* = 3).

### 2.6. Immunostaining

AVIC explant cultures were fixed in 1% paraformaldehyde for 3 mins followed by 4% paraformaldehyde for 10 mins (Affymetrix, 19943) and washed with 1X phosphate buffered saline (PBS) and PBST solution (PBS+0.01% tween 20). Then immunostaining was performed according to the supplied protocol provided by the antibody manufacturer. The following antibodies are used for immunofluorescence (IF): anti-Asporin (1 : 500 dilution, Abcam ab154404) and anti-*α*-Sma (1 : 400 dilution, Thermo Fisher Scientific, 14-9760-82). A rabbit polyclonal secondary antibody (Goat anti-Rabbit IgG H&L, Alexafluor 488, ab150077) was applied against Asporin primary antibodies. All primary antibody incubations were made overnight at 4°C. All cell nuclei were stained with DAPI (SRL, 18668). As negative controls, cultured AVICs were incubated with only the secondary antibody, but with no primary antibody incubation.

### 2.7. Protein Isolation and Western Blotting

Protein lysates were isolated from VICs using ice-cold RIPA lysis buffer (250 mM NaCl, 50 mM Tris pH 7.5, 0.1% SDS, 1% Triton X, and 5 mM EDTA) containing protease inhibitor mixture (Genetix GX2811AR) and phosphatase inhibitor mixture (Genetix GX0211AR) according to the manufacturer's instructions. Western blots were performed as described previously (Evans-Anderson et al., 2008) and the membranes were incubated with secondary antibody (1 : 4000; Abcam ab97051). Immunoblots were developed using chemifluorescent detection with the Clarity Western ECL Substrate reagent (Bio-Rad 1705060) and scanned using ChemiDoc MP (Bio-Rad). Signal intensities were quantified with the ImageJ software (NIH). Asporin has a dilution 2.5 *μ*g/ml (Abcam, ab58741). GAPDH (1 : 2000; BioBharati, BB-AB0060) antibody reactivity was used as a loading control. Statistical significance was determined by Student's *t*-test (*p* < 0.05).

### 2.8. Bright-Field and Fluorescence Microscopy

All light microscopic images were taken using inverted microscope and attached camera (Nikon SMZ800). AVICs in cultures were also being imaged in the bright field using an inverted microscope (Zeiss). For immunostained slides, fluorescence was detected using a microscope (Zeiss lab, flu microscope, and Leica fluorescence microscope) and images were captured using the Zeiss software: ISC capture and Leica software, LAS X.

### 2.9. Alizarin Red S Staining

For Alizarin Red S staining, the cells were washed with 1X cold phosphate buffer saline (PBS) and fixed with 4% paraformaldehyde in PBS for 45 mins at 4°C. For qualitative staining, 2% Alizarin Red S (Sigma) solution was used, and for quantitative analyses, 40 mM Alizarin Red S solution was prepared [5]. After cell fixation, the cells were stained with freshly prepared 2% Alizarin for 30 mins. Then cell plates were washed with distilled water (dH_2_O) 2-3 times for 2 mins each and air-dried. Digital bright-field images were taken using the LAS X software (Leica).

On the other hand, fixed cells were stained with freshly prepared 40 mM Alizarin Red S for 30-40 mins with gentle shaking at room temperature. For quantitative assay, 800 *μ*l of 10% acetic acid (for 60 mm cell plates each) was used to incubate the fixed cells at room temperature for 30 mins. Then, the cell suspensions were collected using a cell scraper into a fresh 1.5 ml microcentrifuge tube. Then these samples were vortexed for 30 seconds followed by heating at 85°C for 10 mins. After that, the samples were carried out for centrifugation at 20000 g for 20 mins. Then, 500 *μ*l of each supernatant was collected in a new tube and 200 *μ*l of 10% ammonium hydroxide was added to each tube to neutralize the acid. pH was measured within 4.1 to 4.5. Finally, the prepared sample solution was aliquoted in 96 well plates (150 *μ*l each) for absorbance. OD values were taken for the control and OS-induced samples using the Gene5 software (BioTek, microplate reader). Then the final quantitative difference was calculated by Student's *t*-test and considered significant for *p* ≤ 0.05 values, where *n* = 3 [22].

### 2.10. Coomassie Blue Staining

To visualize and capture the morphology of isolated adult avian valve interstitial cells (AVICs) in culture, the cells were stained with Coomassie brilliant blue after 4 days of culture. The cultured cells were washed once with 1X PBS and incubated with the Coomassie brilliant blue solution for 30-40 mins. Next, the Coomassie blue stain was removed by washing with dH_2_O for 2-3 times and air-dried before imaging. Then the bright-field images were captured using an inverted microscope (Zeiss).

### 2.11. Statistical Analyses

To derive all the fold changes of gene expression in qRT-PCR data, we have analyzed the normalized expressions using cycle threshold (CT) values in triplicates for both biological and technical replicates for all independent experiments. We have applied Student's *t*-test, specifically (two sided) two-tailed, to observe the statistical significance (*p* ≤ 0.05).

Next, for Asporin immunostaining experiments, all the counted values are taken and after generating the percentages in each set of experiment, graphs have been plotted. Overall, all the statistical analyses have been reported with standard error of mean (SEM) subjected to Student's *t*-test (*p* ≤ 0.05).

Also for Alizarin quantitative assays, OD (optical density) values are considered as absorbance of Alizarin Red stained cell solutions for statistical analyses. Here also, we have applied Student's *t*-test and considered the fold changes significant for *p* ≤ 0.05 values.

### 2.12. Cell Viability Assay

Cell viability was determined post treatment with recombinant Asporin protein (rhAsp) by counting DAPI stained cell nuclei. For this, we have counted the percentage of viable cells (DAPI positive cells) by the following formula: [(Total number of viable cells/Total number of cells seeded during the culture and counted by hemocytometer) × 100].

## 3. Results

### 3.1. Establishment and Characterization of Adult Avian Valve Interstitial Cells (AVICs) in Culture with Marker Gene Expression Analyses

**Figure 1:**
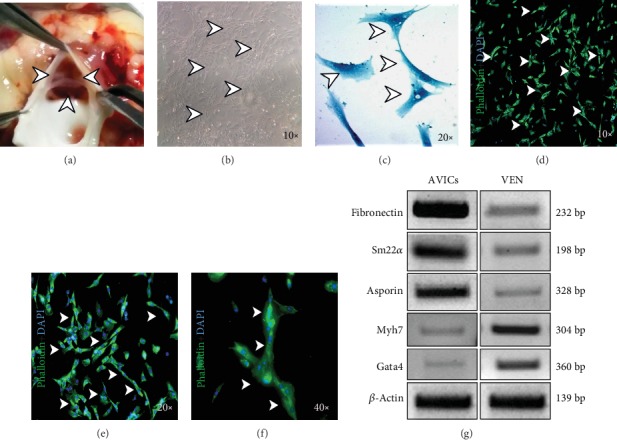
Isolation and characterization of avian adult aortic valve interstitial cells (AVICs) *in vitro*. (a) Light microscopic image of tri-cusps at the basement of adult avian aortic valves (arrowheads). (b) Bright filed image (×10) of primary chick AVICs culture. AVIC cells are characterized with filamentous morphology (shown by arrowheads) after 48-72 hr culture. (c) Bright-field images (×20) of Coomassie brilliant blue-stained AVICs with distinct morphology are taken from cultured avian AVIC cells. (d–f) AVICs were immunostained with *α*-Sma (green), marking myofibroblasts, and counterstained with DAPI (blue) positive nuclei (×10 (d), ×20 (e), and ×40 (f); marked by arrowheads). (g) Gene expression analyses of AVIC-specific markers and *Asporin*, comparing with ventricle (VEN) markers, by RT-PCR. Here, increased expressions of *Fibronectin*, *Sm22α*, and *Asporin* are detected in AVICs compared to the VEN tissue, and in contrast, VEN-specific markers (*Myh7* and *Gata4*) have shown increased expression in the isolated VEN tissue compared to AVIC cells. *β*-Actin was used for equal mRNA input and RT efficiency (*n* = 3).

Several previous reports have identified that the cultured aortic valvular interstitial cells (AVICs) undergo osteoblast differentiation resulting in AVICs mineralization and subsequent calcification [3, 7, 23]. Adult avian whole hearts have been collected and three aortic valvular cusps have been dissected out from each sample. Next AVICs have been isolated from dissected aortic tri-cusps and cultured. The bright-field image has shown three thin and distinct cusps at the base of the aorta (arrowheads in Figure 1(a)). Following the standardized protocol as mentioned in the methods section, the primary AVICs culture has been established successfully. Cells have started to grow and show distinct, clear filamentous morphology after 48-72 hrs of seeding. The bright-field microscopic image has shown AVICs with well-defined spindle-shaped morphology (arrowheads in Figure 1(b)). Next, the Coomassie brilliant blue stain was used to further demonstrate the more distinct morphology of AVICs under higher magnification (arrowheads in Figure 1(c)). To further characterize the isolated AVICs, immunostaining was performed against the *α*-Sma antibody, a known VIC marker, and a strong expression has been detected as shown in Figures 1(d)–1(f) (arrowheads). After 4-5 days of culture, healthy AVICs have been used for further experimentations.

Selective marker gene expression has been determined to validate AVICs isolation.  Figure 1(g) has shown marker gene expression at mRNA level isolated from AVICs in culture compared to the left ventricular tissue dissected out from adult avian hearts used for AVICs isolation. We have performed gene expression analyses of AVIC-specific markers (*Fibronectin* and *Sm22α*), *Asporin* as our molecule of interest along with cardiomyocyte (CM) specific markers (*Myh7* and *Gata4*) by reverse-transcriptase PCR (RT-PCR) on isolated AVICs and ventricular (VEN) tissue from adult avian hearts (Figure 1(g)). In both, 1 *μ*g of total RNA is used as a template to synthesize specific cDNAs for reverse-transcriptase PCR analyses. Two AVIC markers (*Fibronectin* and *Sm22α*) have shown increased expression in AVICs compared to VEN tissue. Also, significant expression of *Asporin* mRNA is detected in AVICs compared to VEN. In contrast, expressions of CM-specific markers *(Myh7* and *Gata4)* increased in the VEN tissue compared to AVICs. Additionally, supplementary III (reverse-transcriptase PCR) data have also shown higher expression of *Asporin* in adult aortic cuspal tissue compared to embryonic outflow tract cushion (OFT) (precursor structure of aortic and pulmonic valves in developing embryo) tissue. Therefore, all these characterization and gene expression data have shown successful establishment of primary avian adult AVICs in culture.

### 3.2. Osteogenic Induction in AVICs in Culture

**Figure 2:**
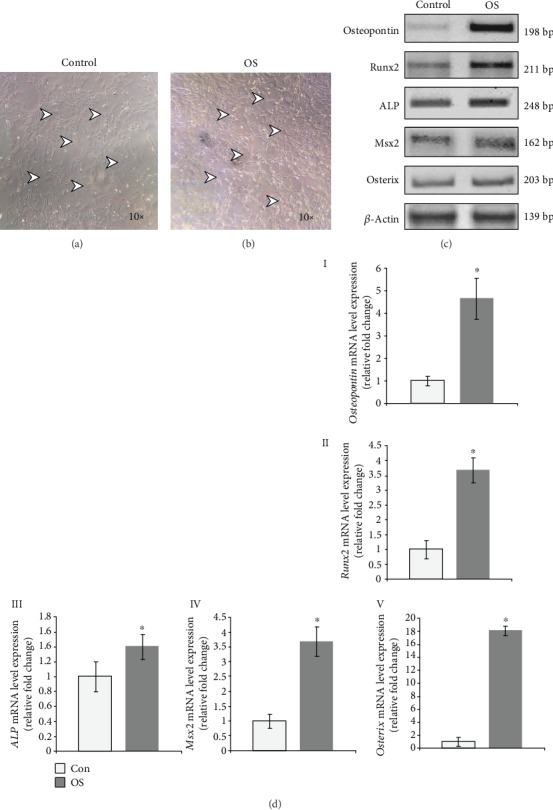
Osteogenic induction in avian adult aortic valve interstitial cells (AVICs) *in vitro*. (a, b) Representative bright-field images of uninduced control (a, marked by arrowheads) and osteogenic (OS) media-induced AVICs in culture (b, marked by arrowheads). (c) Gene expressions of osteogenic markers (*Osteopontin*, *Runx2*, *ALP*, *Msx2*, and *Osterix*), by reverse-transcriptase PCR, have shown elevated expression in OS-induced AVICs compared to control normal AVICs. *β*-Actin was used for equal mRNA input and RT efficiency. (d) (I–V): relative mRNA level expressions have been further validated by real-time PCR. *Osteopontin*, *Runx2*, *ALP*, *Msx2*, and *Osterix* all increased by 3.65 fold, 2.68 fold, 0.4 fold, 2.68 fold, and 17.12 fold, respectively. *β*-Actin was used to analyze normalized gene expressions. Statistical significance was determined by Student's *t*-test, where ^∗^ denotes *p* ≤ 0.05 and *n* = 3.

After successful establishment of AVICs and to induce osteogenesis *in vitro*, AVICs were treated with osteogenic (OS) induction media for 96 hrs. Complete media was supplemented with OS induction ingredients (*β*-glycerophosphate (10 mM), L-ascorbic acid (10 nM), and dexamethasone (0.1 *μ*M)) as described by others previously [9]. The bright-field images of both control and OS-treated healthy AVICs cultures are shown in Figures 2(a) and 2(b) (arrowheads). Next, as expected, the RT-PCR data have indicated that several osteogenic marker-specific gene (*Osteopontin*, *Runx2*, *ALP*, *Msx2*, and *Osterix*) expressions have been significantly upregulated upon OS treatment compared to untreated control AVICs (Figure 2(c)).

Likewise, consistent with the RT-PCR data, mRNA level expressions of *Osteopontin*, *Runx2*, *ALP*, *Msx2*, and *Osterix* genes have shown increased by 3.65, 2.68, 0.4, 2.68, and 17.12 folds, respectively, determined by quantitative real-time PCR (qRT-PCR) analyses in OS-treated AVICs compared to untreated controls (Figure 2(d), I–V). Overall, these data have confirmed that osteogenic induction with OS media resulted in increased expression of several osteogenic markers in AVICs. Thus, these data show successful osteogenic induction in adult AVICs in culture.

### 3.3. Reduced Expression of Endogenous Asporin in Cultured AVICs upon Osteogenic Induction

**Figure 3:**
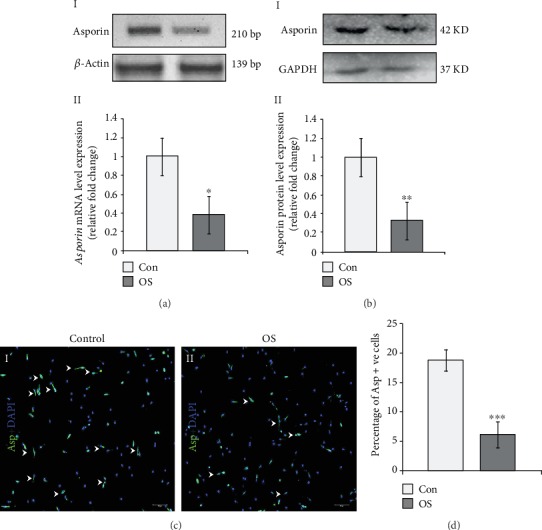
Endogenous Asporin mRNA and protein expressions decrease upon osteogenic induction in avian adult aortic valve interstitial cells (AVICs) *in vitro*. (a) (I and II): reduced *Asporin* gene expression in osteogenic (OS) media-induced AVICs compared to uninduced control AVICs, by reverse-transcriptase PCR and real-time PCR. Here, *Asporin* has shown reduced expression in OS-treated AVICs compared to control AVICs (a, I). Also, quantitative expression data reveals 0.62 fold decreased expression of *Asporin* in OS-treated AVICs compared to control AVICs (a, II). (b) (I and II): reduced Asporin protein expression in osteogenic (OS) media-induced AVICs compared to uninduced control AVICs, by western blot analyses. Here, Asporin has shown decreased expression in OS-treated AVICs compared to control AVICs, according to band intensity (b, I). Also, quantitative expression data reveals 0.67 fold decreased expression of Asporin in OS-treated AVICs compared to control AVICs (*p* = 0.008). (c) (I and II): here, AVICs were immunostained with Asporin antibody (green) and nuclei were counterstained with DAPI (blue), showing decreased number of Asp+ cells in OS-treated AVICs compared to control AVICs (marked by arrowheads). (d) Here, quantitative data demonstrate that OS-treated AVICs show decreased number of Asporin-positive cells as detected by 12.65% compared to untreated control AVICs. Statistical significance was determined by Student's *t*-test, where ^∗^ denotes *p* ≤ 0.05 and ^∗∗∗^ denotes *p* ≤ 0.001 and *n* = 3.

After successful establishment and osteogenic induction of adult AVICs in cultures, the *Asporin* mRNA expression has been analyzed by RT-PCR data with 1 *μ*g of the total RNA template, used to synthesize cDNA. In addition, the Asporin protein expression has also been analyzed in control and OS-treated AVICs in culture. Interestingly, the decreased level of *Asporin* mRNA is detected in OS-induced AVICs compared to untreated controls as performed by both RT-PCR data and qRT-PCR assays. In Figure 3(a), I, the RT-PCR data indicate that *Asporin* expression is markedly downregulated in OS-treated AVICs compared to control AVICs as per the nucleic acid agarose gel band intensity. Likewise, consistent with reverse-transcriptase PCR data, *Asporin* mRNA expression is decreased by 0.62 fold in OS-treated AVICs compared to untreated controls (Figure 3(a), II).

Also at protein level, Asporin has been found to decrease also in OS-treated AVICs compared to control AVICs. In Figure 3(b), I and II, the western blot data show that Asporin has downregulated by 0.67 fold (*p* = 0.008) in OS-treated AVICs compared to control protein.

Additionally, to further validate the result of osteogenic induction and both *Asporin* mRNA and protein expressions, OS-treated AVICs are immunostained with Asporin- (Asp-) specific antibody. Consistent with the mRNA expression data, a decreased number of Asporin-positive (Asp^+^) cells (arrowheads in Figure 3(c), I) are detected in OS-treated AVICs compared to the number in untreated control AVICs (arrowheads in Figure 3(c), II). The quantitative cell count data further reveal around 12.65% decrease in Asporin-labeled cells in OS-treated AVICs compared to untreated controls, respectively (Figure 3(d)). A minimum of 500 DAPI^+^ nuclei are counted in each set of experiments (*n* = 3).

Overall, these data suggest that the osteogenic induction decreases endogenous *Asporin* expression both at mRNA and protein levels in AVICs in culture.

### 3.4. Marker Gene Expression Analyses in AVICs upon Induction with Osteogenic (OS) Media

**Figure 4:**
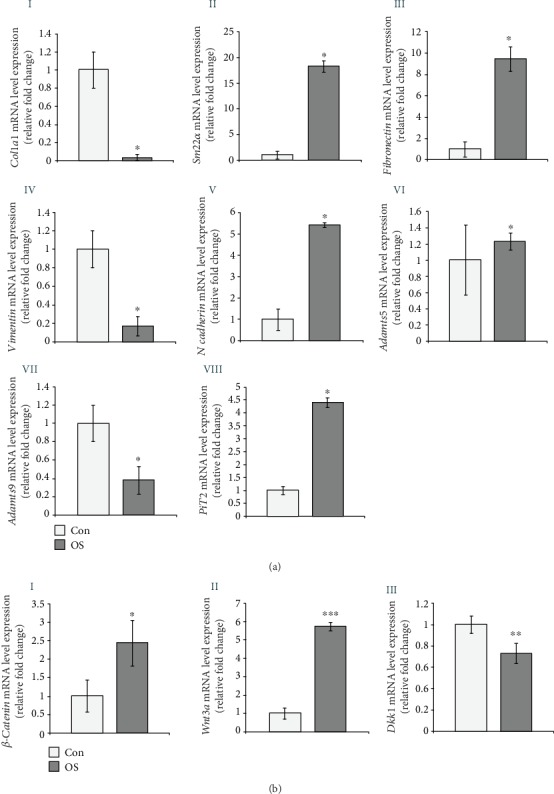
Selective AVICs marker gene expression analyses after osteogenic (OS) media induction in avian adult AVICs *in vitro*. (a) (I–VII): qRT-PCR data show that *Col1a1*, *Vimentin*, and *Adamts9* have been downregulated by 0.97 fold, 0.83 fold, and 0.62 fold, respectively (I, IV, and VII). In addition, *Sm22α*, *Fibronectin*, *N-cadherin*, *Adamts*5, and *PiT2* have been upregulated by 17.25 fold, 8.51 fold, 4.42 fold, 0.23 fold, and 3.36 fold, respectively (II, III, V, VI, and VIII). (b) (I–III): similarly, positive regulators of Wnt signaling, *β-catenin* and *Wnt3a*, have been upregulated by 1.44 fold and 4.73 fold, respectively (I and II). In contrast, the negative modulator of Wnt signaling, *Dkk1* mRNA, is decreased by 0.27 fold (III). Statistical significance was determined by Student's *t*-test, where ^∗^ denotes *p* ≤ 0.05, ^∗∗^ denotes *p* ≤ 0.01 and ^∗∗∗^ denotes *p* ≤ 0.001 and *n* = 3.

Next, both control and OS-treated AVICs have been taken, and additional selective marker gene expression has been determined. Expression of several extracellular matrix (ECM) and Wnt/*β*-catenin signaling pathway-specific genes sensitive to osteogenic induction is determined by qRT-PCR analyses. Expression of *Col1a1*, an ECM marker, has been significantly downregulated by 0.97 fold in OS-treated, compared to control AVICs (Figure 4(a), I). A myofibroblast marker, *Sm22α* increased by 17.25 fold in OS-treated, compared to control AVICs (Figure 4(a), II). Another ECM marker, *Fibronectin*, has been upregulated by 8.51 fold (Figure 4(a), III), intermediate filament marker, *Vimentin*, has decreased by 0.83 fold (Figure 4(a), IV), cell adhesion marker *N-cadherin* has increased by 4.42 fold in OS-treated compared to control AVICs (Figure 4(a), V). Two matrix metalloproteinases (MMPs) Adamts5 and Adamts9 have increased by 0.23 fold (Figure 4(a), VI) and decreased by 0.62 fold (Figure 4(a), VII), respectively, in OS-treated AVICs compared to control AVICs in culture. Interestingly, the decreased mRNA expression level of collagen (*Col1a1*) and valvular interstitial cell marker (*Vimentin*) have also been indicative of activated cell phenotypes with induced osteogenesis. Also, we have detected increased (3.36 fold) (Figure 4(a), VIII) expression of *PiT2* mRNA, indicative of intracellular phosphate level in OS-induced AVICs compared to control. But we could not able to detect the expression of *PiT1* transcript in our OS-induced AVICs in culture. Overall, several ECM genes responsible for the pathological remodeling have been altered significantly towards mineralization and calcific phenotypes in cultured AVICs induced with osteogenesis.

Furthermore, in OS-treated AVICs in culture and consistent with previous report [9], genes/proteins involved in Wnt/*β*-catenin signaling including but not limited to *β-catenin*, *Wnt3a*, and *Dkk1* mRNA have shown altered expression compared to untreated controls. In OS-treated AVICs, the qRT-PCR data show increased expression of *β-catenin* and *Wnt3a* mRNA (1.44 and 4.73 fold, respectively) and decreased expression of *Dkk1* mRNA (0.27 fold), compared to untreated controls (Figure 4(b), I–III).

In addition, BMP/Smad signaling- and Notch/Sox9 signaling-specific gene markers have also been compared between control and OS-treated AVICs (Supplementary figure IV). Here, BMP2 has increased by 0.07 fold, Smad1 has decreased by 0.15 fold, and Smad5 and Smad8 increased by 7.51 and 6.11 folds in OS-treated AVICs compared to untreated control cells, respectively. Likewise, *Notch1* mRNA has been increased by 1.62 fold and *Sox9* mRNA has decreased by 0.67 fold in OS-treated compared to untreated AVICs in culture. Thus, other signaling pathway genes, involved in valvular osteogenesis, also get significantly altered upon OS induction on AVICs (Supplementary figure IV).

Interestingly, earlier reports have shown that Sox9 directs chondrogenic differentiation by blocking osteogenic differentiation [24, 25]. Here, we have found that *Sox9* mRNA expression is reduced after osteogenic induction in AVICs. In contrast, we also show that (Figure 2) *Runx2* mRNA expression increased in OS-induced AVICs, indicating Runx2 involvement in osteogenesis process. Taken together, others and our data might suggest that the balance between these two transcription factors is important for osteogenic induction in AVICs *in vitro*.

Overall, all these data show altered expression of several AVICs markers, ECM genes, and multiple signaling pathways sensitive to OS induction in AVICs in culture including Wnt/*β*-catenin signaling.

All the results of gene expression analyses showing changes between control and OS-induced AVICs are being represented as a list in Table 2.

### 3.5. Overexpression of Asporin Reduces Mineralization on AVICs Maintained in Osteogenic Medium

**Figure 5:**
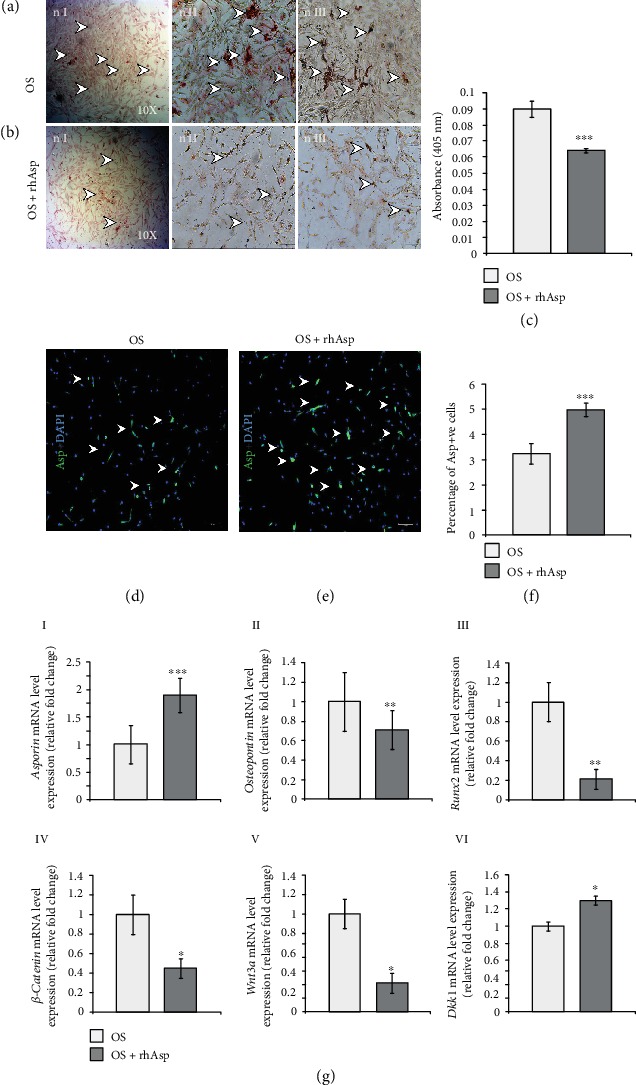
Human recombinant Asporin (rhAsp) treatment decreases matrix mineralization in osteogenic (OS) media-induced avian adult AVICs *in vitro*. (a, b) Alizarin Red S staining data is showing (a, nI–nIII) bright-field microscopic images (nI (×10) and nII and nIII (×16)) of OS-induced AVICs (arrowheads) and (nI–nIII) bright-field microscopic images (nI (×10) and nII and nIII (×16)) of rhAsp-treated OS-induced AVICs (arrowheads). (c) Quantitative assay of Alizarin Red S shows that absorbance of rhAsp-treated OS-induced AVICs decreased (0.064) compared to only OS-induced AVICs (0.089) (*p* ≤ 0.05, *n* = 3). (d, e) Here, immunostaining data show that a number of Asporin-positive (Asp^+^) cells increased in rhAsp-treated OS-induced AVICs compared to OS-induced AVICs (arrowheads). (f) Quantitative data show 1.75% increase in Asp^+^ cells in rhAsp-treated OS-induced AVICs compared to OS-induced AVICs only. (g) (I and VI): qRT-PCR data show that *Asporin* is upregulated by 0.9 fold (I), osteogenic markers *Osteopontin* and *Runx2* are downregulated by 0.29 fold (II) and 0.79 fold (III), and Wnt signaling-specific markers, *β-catenin* and *Wnt3a*, are downregulated by 0.55 fold (IV) and 0.71 fold (V), whereas *Dkk1* is upregulated by 0.3 fold (VI), respectively, in rhAsp-treated OS-induced AVICs compared to OS-induced AVICs only. Statistical significance was determined by Student's *t*-test, where ^∗^ denotes *p* ≤ 0.05, ^∗∗^ denotes *p* ≤ 0.01, and ^∗∗∗^ denotes *p* ≤ 0.001 and *n* = 3.

Next, to determine the effect of Asporin protein on AVICs mineralization, OS-induced cells have been also treated with Asporin recombinant protein (rhAsp, 150 ng/ml) for 24 hrs to assess both qualitative and quantitative mineralization by Alizarin Red S staining. Asporin protein localization has also been assessed by immunostaining to compare the expression between OS- and OS+rhAsp-treated AVICs in culture. Also, to confirm changes in marker gene expression upon rhAsp treatment, qRT-PCR was performed. AVICs, which have been treated with OS induction media for 3 days, show increased mineralization by Alizarin Red S staining (Figure 5(a), arrowheads in nI–nIII). Interestingly, comparing with this OS control cells in Figure 5(a), a reduced level of mineralization has been observed in Figure 5(b) (arrowheads in nI–nIII) after treatment with rhAsp. Next, this data have been further confirmed with alizarin quantitative assay in Figure 5(c). This quantitative data indicate decreased absorbance (OD) (0.089) of alizarin-stained rhAsp-treated AVICs compared to OS control (0.064) without any rhAsp. Next, Figures 5(d) and 5(e) have shown the difference of Asporin- (Asp-) positive AVICs between OS control and rhAsp-treated AVICs. As expected, it shows increased numbers of Asporin-positive AVICs in rhAsp-treated cells (Figure 5(e)) compared to OS controls alone (Figure 5(d)). This is further validated by analyzing and quantifying the increased percentage (1.75%) of Asporin-positive AVICs in OS+rhAsp compared to OS control only (Figure 5(f)).

Furthermore, the mRNA expression level of related gene makers is also evaluated on OS control and rhAsp-treated AVICs by qRT-PCR (Figure 5(g), I–VI). These data have detected that *Asporin* mRNA has been significantly upregulated by 0.9 fold; both osteogenic gene markers (*Osteopontin* and *Runx2*) have decreased by 0.29 fold and 0.79 fold, respectively, in rhAsp-treated AVICs compared to OS control only. Also, positive regulators of Wnt signaling (*β-catenin*, *Wnt3a*) both decreased by 0.55 fold and 0.8 fold, respectively, in rhAsp-treated AVICs compared to OS control only. In contrast, expression of *Dkk1*, a negative regulator of Wnt signaling, has increased by 0.3 fold in rhAsp-treated AVICs compared to OS control only. Hence, all these data clearly have shown that Asporin significantly inhibits valvular mineralization induced with OS induction media.

In addition, to determine the effect of rhAsp protein on AVICs viability, cell viability assay has been performed. Likewise, we have used three concentrations (100 ng/ml, 150 ng/ml, and 200 ng/ml) of rhAsp protein in a dose-dependent manner in AVICs in culture and as shown by the Supplementary figure V. This data have shown that 88%, 81.21%, 93.81%, and 76.7% viable cells were observed in control, 100 ng/ml-, 150 ng/ml-, and 200 ng/ml-treated AVICs, respectively (Supplementary figure V A-D). No statistical significance is observed in AVICs viability across all the tested concentrations of rhAsp protein (Supplementary figure V E). The total number of DAPI positive nuclei have been counted in each group with minimum of 500 nuclei per group (*n* = 3).

Together, these data strongly suggest that recombinant Asporin protein significantly reduces the level of mineralization in adult avian AVICs maintained in OS induction media *in vitro*.

All the results of gene expression analyses, showing changes between OS control and OS+rhAsp-treated AVICs, are being represented as a list in Table 3.

### 3.6. Human Recombinant Asporin Protein Inhibits AVICs Mineralization Induced with Osteogenic Media and Wnt Signaling Activation

**Figure 6:**
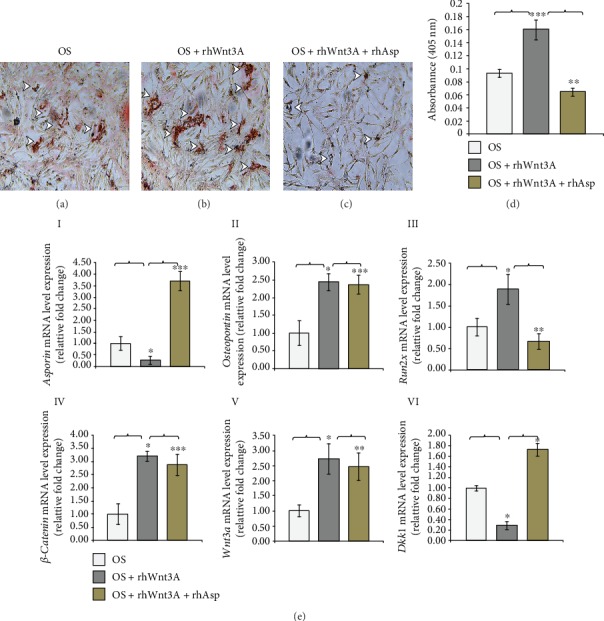
Human recombinant Asporin (rhAsp) treatment decreases matrix mineralization in recombinant Wnt3a- (rhWnt3A-) treated osteogenic (OS) media-induced avian adult AVICs in culture. (a–c) Bright-field microscopic images (×10) of alizarin-stained OS-induced AVICs show increased matrix mineralization in rhWnt3a-treated OS-induced AVICs compared to OS-induced AVICs. Moreover, decreased matrix mineralization is detected in rhWnt3A+rhAsp-treated OS-induced AVICs (arrowheads). (d) Quantitative assay of Alizarin Red S shows that absorbance of rhWnt3A-treated OS-induced AVICs is upregulated (0.16) compared to only OS-induced AVICs (0.093), and absorbance of rhWnt3A+rhAsp-treated OS-induced AVICs is downregulated (0.065) compared to rhWnt3A-treated OS-induced AVICs (*p* ≤ 0.05, *n* = 3). (e) (I and VI): here, qRT-PCR data show that *Asporin* decreased by 0.73 fold in rhWnt3A-treated and increased by 3.43 fold in rhWnt3A+rhAsp OS-induced AVICs (I). *Osteopontin* and *Runx2* mRNA expressions increased by 1.44 fold and 0.89 fold in rhWnt3A-treated and decreased by 0.07 fold and 1.23 fold in rhWnt3A+rhAsp-treated OS-induced AVICs (II and III). *β-Catenin* and *Wnt3A* mRNA increased by 2.2 fold and 1.73 fold in rhWnt3A-treated and decreased by 0.33 fold and 0.26 fold in rhWnt3A+rhAsp OS AVICs. Also, *Dkk1* decreased by 0.71 fold in rhWnt3A-treated and increased by 1.44 fold in rhWnt3A+rhAsp-treated OS-induced AVICs. Statistical significance was determined by Student's *t*-test, where ^∗^ denotes *p* ≤ 0.05, ^∗∗^ denotes *p* ≤ 0.01, and ^∗∗∗^ denotes *p* ≤ 0.001 and *n* = 3.

Next, to determine the effect of recombinant Asporin protein on AVICs mineralization maintained both in OS induction media and with increased level of Wnt signaling, AVICs have been further treated with Wnt3a recombinant protein (rhWnt3a, 150 ng/ml, 24 hrs) to activate Wnt signaling in addition to OS induction. To our surprise, recombinant Asporin protein (rhAsp, 150 ng/ml, 24 hrs) inhibits mineralization of AVICs supplemented with OS+rhWnt3a compared to AVICs maintained in OS+rhWnt3a alone. Likewise, Figures 6(a)–6(c) have shown Alizarin-stained osteogenic media-induced AVICs (OS) culture for 6 days followed by 24 hr treatments with either rhWnt3a or rhWnt3a+rhAsp. The basal levels of mineralization have been detected and marked qualitatively in Figure 6(a) (arrowheads). Compared with this OS induction alone, an increased level of mineralization has been detected in OS+rhWnt3a-treated AVICs (arrowheads in Figure 6(b)). Interestingly, the mineralization level has decreased when OS+rhWnt3a AVICs have been further treated with rhAsp as evident by a reduced level of Alizarin reactivity (arrowheads in Figure 6(c)). These qualitative data are further validated by quantitative assay in Figure 6(d). Increased absorbance (OD) of alizarin intensity (0.160) has been shown in OS+rhWnt3a-treated AVICs compared to OS induction alone (0.093). Interestingly, decreased absorbance (OD) of alizarin has been detected when OS+rhWnt3a-treated AVICs were further treated with rhAsp protein (0.065). Overall, addition of Asporin protein inhibits OS media-induced AVICs mineralization as detected by Alizarin Red S staining, both qualitatively and quantitatively.

Next, to determine the mRNA expression level of the indicated genes involved in osteogenesis and Wnt/*β*-catenin signaling including Asporin, detected in OS-, OS+rhWnt3a-, and OS+rhWnt3a+rhAsp-treated AVICs, qRT-PCR assays were used (Figure 6(e), I–VI). *Asporin* mRNA has been significantly downregulated in rhWnt3a-treated and upregulated in rhWnt3a+rhAsp-treated OS-induced AVICs. It has decreased by 0.73 fold in rhWnt3a-treated AVICs compared to OS control and again increased by 3.43 fold in rhWnt3A+rhAsp-treated OS-induced AVICs compared to rhWnt3a-treated AVICs. Likewise, expression of osteogenic markers, *Osteopontin* and *Runx2*, has been both increased in rhWnt3a-treated and decreased in rhWnt3a+rhAsp-treated OS-induced AVICs. *Osteopontin* mRNA expression has been increased by 1.44 fold in rhWnt3a-treated compared to OS control and decreased by 0.07 fold in rhWnt3a+rhAsp-treated OS-induced AVICs compared to only rhWnt3a-treated AVICs. Likewise, *Runx2* mRNA expression has increased by 0.89 fold in rhWnt3A-treated compared to OS control and decreased by 1.23 fold in rhWnt3a+rhAsp OS AVICs compared to only rhWnt3A-treated AVICs. Wnt pathway modulators, *β-catenin* and *Wnt3a* mRNA expressions, have been also determined in both treatments on OS-induced AVICs. *β-Catenin* mRNA has increased by 2.2 fold in rhWnt3a-treated compared to OS control and decreased by 0.33 fold in rhWnt3a+rhAsp-treated OS-induced AVICs compared to only rhWnt3a-treated AVICs. Similarly, *Wnt3A* has increased by 1.73 fold in rhWnt3A-treated compared to OS control and decreased by 0.26 fold in rhWnt3A+rhAsp-treated OS-induced AVICs compared to only rhWnt3a-treated AVICs. In contrast, mRNA expression of a negative regulator of Wnt signaling, *Dkk1* has decreased by 0.71 fold in rhWnt3a-treated compared to OS control and increased by 1.44 fold in rhWnt3A+rhAsp-treated OS-induced AVICs compared to only rhWnt3a-treated AVICs. Thus, these gene expression data are consistent with the mineralization status of OS-induced AVICs with or without treatments.

Together, all these data strongly suggest that recombinant Asporin protein inhibits mineralization in OS-induced AVICs in culture along with increased mRNA level of Wnt/*β*-catenin signaling-specific gene expressions.

All the results of gene expression analyses showing changes between OS control, OS+rhWnt3a-, and OS+rhWnt3a+rhAsp-treated AVICs are represented as a list in Table 4.

### 3.7. Inhibition of Wnt Signaling Fails to Rescue OS-Induced AVICs Mineralization *In Vitro*

**Figure 7:**
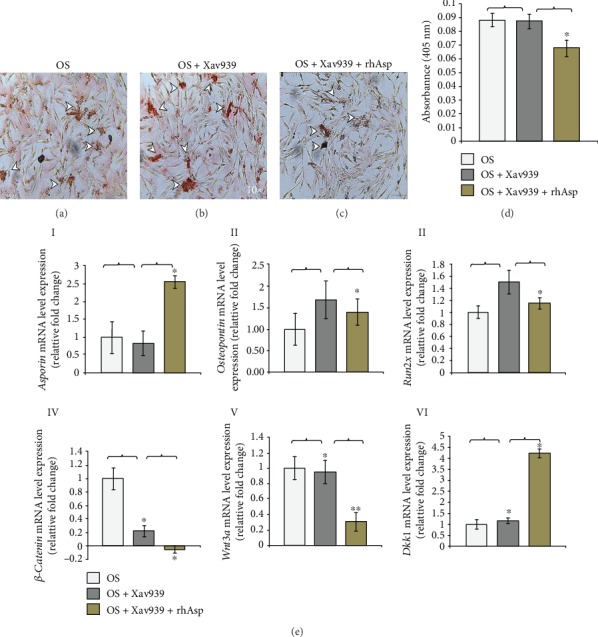
Human recombinant Asporin (rhAsp) treatment also decreases matrix mineralization in Xav939-treated osteogenic (OS) media-induced avian adult AVICs in culture. (a–c) Bright-field microscopic images (×10) of alizarin stained OS-induced AVICs show unchanged matrix mineralization in Xav939-treated OS-induced AVICs compared to OS-induced AVICs only. Moreover, decreased matrix mineralization is detected in Xav939+rhAsp-treated OS-induced AVICs (arrowheads). (d) Quantitative assay of Alizarin Red S shows that the absorbance of Xav939-treated OS-induced AVICs is similar (0.089) compared to only OS-induced AVICs (0.088), and the absorbance of rhWnt3A+rhAsp-treated OS-induced AVICs decreased (0.068) compared to Xav939-treated OS-induced AVICs (*p* ≤ 0.05, *n* = 3). (e) (I and VI): qRT-PCR data show that *Asporin* is unchanged in both OS-induced AVICs and Xav939-treated AVICs, whereas it increased by 1.71 fold in Xav939+rhAsp OS-induced AVICs (I). *Osteopontin* and *Runx2* mRNA expressions are unchanged in both OS-induced AVICs and Xav939-treated AVICs, whereas these decreased by 0.28 fold and 0.35 fold in Xav939+rhAsp OS-induced AVICs, respectively (II and III). *β-Catenin* decreased by 0.78 fold and 0.27 fold in Xav939-treated and in Xav939+rhAsp-treated OS-induced AVICs, respectively (IV). Similarly, *Wnt3A* decreased by 0.05 fold and 0.65 fold in Xav939-treated and inXav939+rhAsp-treated OS-induced AVICs, respectively (V). Also, *Dkk1* increased by 0.18 fold and 3.09 fold inXav939-treated and in Xav939+rhAsp-treated OS-induced AVICs, respectively. Statistical significance was determined by Student's *t*-test, where ^∗^ denotes *p* ≤ 0.05 and ^∗∗^ denotes *p* ≤ 0.01 and *n* = 3.

Next, to determine if Wnt signaling is sufficient to induce AVICs mineralization in culture, OS-induced AVICs have been treated with Wnt signaling inhibitor Xav939 (2 *μ*M) to inhibit Wnt signaling and also treated with recombinant human Asporin protein (rhAsp, 150 ng/ml) was used for treatment for 24 hrs in both cases. Assessments of qualitative and quantitative mineralization have been performed by Alizarin Red S staining of OS-induced and treated AVICs. The mRNA expression levels of several gene markers have been analyzed for Xav939 (Wnt signaling inhibitor) and Xav939+rhAsp-treated OS-induced AVICs by qRT-PCR assays. Figures 7(a)–7(c) show Alizarin-stained OS-induced AVICs maintained for 6 days in culture followed by 24 hr treatment of either Xav939 or Xav939+rhAsp protein. Basal levels of mineralization have been detected and marked qualitatively by arrowheads in Figure 7(a). Interestingly, detection of unchanged mineralization status in Xav939-treated OS-induced AVICs (arrowheads in Figure 7(b)), compared to OS induction alone in Figure 7(a), indicates the presence of other active signaling pathway(s) promoting mineralization in OS-induced AVICs treated with Xav939. But, as expected, treatment of rhAsp inhibits mineralization in Xav939-treated OS-induced AVICs (arrowheads in Figure 7(c)) compared to Xav939 treatment alone (arrowheads in Figure 7(b)). Further, absorbance (OD) data generated from alizarin-stained AVICs intensity are further validated by quantitative assay in Figure 7(d). Consistent with the qualitative data, alizarin absorbance of OS-induced AVICs and Xav939-treated OS-induced AVICs are 0.089 and 0.88 (ODs), respectively. But in both Xav939- and rhAsp-treated OS-induced AVICs, it further decreased to 0.68 (OD), indicative of reduced level of mineralization.

Next, to determine the gene expression changes in OS induction alone, Xav939-treated OS-induced and Xav939+rhAsp-treated OS-induced AVICs, qRT-PCR analyses have been done (Figure 7(e), I–VI). Consistent with the mineralization data, *Asporin* mRNA expression has been unchanged in Xav939-treated OS-induced but significantly upregulated in Xav939+rhAsp-treated OS-induced AVICs. Likewise, *Asporin* mRNA has increased by 1.71 fold in Xav939+rhAsp-treated OS-induced AVICs compared to Xav939-treated OS-induced AVICs. In addition, expressions of osteogenic markers (*Osteopontin* and *Runx2*) have been unchanged in Xav939-treated OS-induced AVICs but significantly downregulated in Xav939+rhAsp-treated OS-induced AVICs. *Osteopontin* and *Runx2* mRNA have decreased by 0.28 fold and 0.35 fold in Xav939+rhAsp OS-induced AVICs, respectively, compared to only Xav939-treated OS-induced AVICs. In addition, positive regulators of Wnt pathway genes, *β-catenin* and *Wnt3A* mRNA expressions, have also been analyzed in both treated groups compared to OS-induced AVICs only. Expression of *β-catenin* mRNA has decreased by 0.78 fold in Xav939-treated AVICs compared to OS control and decreased by 0.27 fold in Xav939+rhAsp-treated OS-induced AVICs compared to only Xav939-treated OS-induced AVICs. Similarly, expression of *Wnt3A* mRNA has decreased by 0.05 fold in Xav939-treated AVICs compared to OS control and decreased 0.65 fold in Xav939+rhAsp-treated OS-induced AVICs compared to only Xav939-treated OS-induced AVICs. In contrast, the negative regulator of Wnt signaling pathway gene, *Dkk1* expression, has been increased by 0.18 fold in Xav939-treated AVICs compared to OS control and increased 3.09 fold in Xav939+rhAsp-treated OS-induced AVICs compared to only Xav939-treated OS-treated AVICs.

Therefore, the presence of mineralization in OS-induced AVICs with a decreased level of Wnt/*β*-catenin signaling further indicates the presence of other active pathway responsible of AVICs mineralization. But, to our surprise, inhibition of mineralization by treatment with rhAsp in Xav939-treated OS-induced AVICs indicates the potential Asporin function independent of multiple osteogenic-promoting signaling pathways to inhibit AVICs mineralization *in vitro*.

Together, all these data strongly suggest that Asporin inhibits mineralization and probably acts upstream of multiple osteogenic inducing pathway genes at mRNA level including Wnt/*β*-catenin signaling in AVICs in culture.

All the results of gene expression analyses showing changes between OS control, OS+Xav939-, and OS+Xav939+rhAsp-treated AVICs are represented as a list in Table 5.

## 4. Discussion

Adult aortic valvular interstitial cell mineralization and active calcification best characterizes the CAVD phenotype in the adult heart. But being expressed in adult aortic valvular cusps, Asporin function in CAVD progression and pathogenesis is unexplored. Therefore, in this report, we have shown for the first time that Asporin might have an inhibitory function in aortic valvular interstitial cell matrix mineralization and calcification with potential therapeutic implication.

Here, we have detected the strong expression of *Asporin* mRNA in adult avian aortic cusps compared to the lower level of expression in embryonic outflow tract cushion (aortic valve precursor) tissue. We have also successfully established an avian adult aortic valve interstitial cell (AVIC) culture system (Figure 1). Due to inability to maintain these isolated primary AVICs in osteogenic medium to induce mineralization for more than 3 days *in vitro*, we could not detect any Von Kossa reactive cells in culture (data not shown), which would further indicate the end-stage osteogenic process with calcium salt deposits. Upon induction of osteogenesis, we have analyzed and detected increased expression of several osteogenic gene markers along with increased levels of mineralization (Figure 2). Osteogenic induction resulted in a decreased level of endogenous Asporin expression with a concomitant increased level of Wnt/*β*-catenin signaling compared to uninduced control AVICs in culture (Figures 3 and 4).

Several reports have identified Wnt/*β*-catenin signaling as a critical modulator for cardiogenesis including cell proliferation, lineage differentiation, and heart morphogenesis including the aortic valve calcification process [19, 26–28]. Therefore, to better understand the associated signaling which promotes mineralization in osteogenic media-induced AVICs in culture, we have manipulated Wnt/*β*-catenin signaling to determine the Asporin function as an antimineralization ECM protein. Interestingly, after osteogenic induction, direct addition of human recombinant Asporin with or without Wnt signaling activation reduces AVICs mineralization *in vitro* (Figures 5 and 6). For mineralization assessments, we have done both qualitative and quantitative assays of Alizarin Red S staining on AVICs in culture. Interestingly, addition of recombinant human Asporin significantly rescues mineralization in AVICs treated with both osteogenic media and Xav939. Together, these data strongly suggest that Asporin inhibits AVICs mineralization in culture induced by either osteogenic induction media alone or in combination with recombinant human Wnt3a protein. In contrast, direct addition of Wnt signaling inhibitor Xav939 does not rescue mineralization significantly compared with osteogenic induction media alone, indicative of additional active pathway promoting mineralization in culture (Figure 7). Consistent with these data, our supplementary figure II has also shown the altered expression of *BMP-Smad*, *Notch1*, and *Sox9* genes in OS-induced AVICs compared to uninduced cells in culture.

Therefore, as hypothesized, Wnt signaling activation synergistically promotes mineralization in AVICs in culture induced with osteogenesis. But, direct addition of recombinant Asporin protein inhibits mineralization. Overall, all these data are highly suggestive that Asporin likely binds directly to Wnt signaling receptors to prevent Wnt ligand binding to competitively inhibit Wnt/*β*-catenin signaling activation and therefore the subsequent osteogenic gene induction. Likewise, a previous report has also demonstrated direct binding of Asporin with Bmp receptor via leucine-rich motif, to inhibit Bmp-Smad1/5/8 signaling for osteogenic induction and subsequent mineralization and calcification in periodontal cells (MPDL22) *in vitro* [16]. Therefore, like Bmp-Smad1/5/8 signaling, Asporin also inhibits Wnt/*β*-catenin signaling to inhibit osteogenic gene induction in OS-treated adult aortic valve cells in culture. Importantly, as of yet, there is no functional crosstalk between Asporin and Wnt/*β*-catenin signaling in connection to AVICs mineralization and calcification.

Overall, Asporin inhibits adult AVICs mineralization induced with either osteogenic induction media alone or in combination with the Wnt signaling activation. Therefore, in this report, we have identified Asporin as a novel antimineralization molecule, which would probably bind directly to receptors of multiple osteogenic promoting pathways to competitively inhibit respective ligand and receptor interaction and subsequent signaling activation to induce osteogenic gene programme in adult AVICs in culture.

## Figures and Tables

**Table 1:** Designed primers for qRT-PCR (IDT PrimerQuest tool).

Serial No.	Genes	Annealing temperature (°C)	Product size (bp)	Primer sequences
	***Chick***			
1	*Asporin*	55.4	328	F: 5′-GCAACATTCCACCAGATAC-3′ R: 5′-AGAGGATTTGCACTCATTTC-3′
2	*Myh7*	53.8	156	F: 5′-CCCCTCAATGAGACAGTGGT-3′ R: 5′-GGCTGAGACAGTCTGGAAGG-3′
3	*Gata4*	59.2	360	F: 5′-GTGTCACCTCGCTTCTCCTT-3′ R: 5′-GTGCCCTGTGCCATCTCT-3′
4	*Col1a1*	52.6	173	F: 5 ′-GTACCTCAGCAAGAACCCCA-3′ R: 5′-AGTGGTAGGTGACGTTCTGG-3′
5	*Sm22α*	52.6	198	F: 5′-ATGTTCCAGACCGTTGACCT-3′ R: 5′-GCCAATGATGTTCTTGCCCT-3′
6	*Vimentin*	53.8	201	F: 5′-CCAGATGCGTGAAATGGAGG-3′ R: 5′-CTCTCTTCTCCCTCCAGCAG-3′
7	*Fibronectin*	56.9	232	F: 5′-AGATGTTCCAAGGGACCTGG-3′ R: 5′-TCCACGACCAGTTACAGCAT-3′
8	*Alp*	53.8	248	F: 5′-GCTAAGGATGAAGGCAAGGC-3′ R: 5′-TACTCCACATCGCTGGTGTT-3′
9	*N-Cadherin*	57.6	219	F: 5′-TTGGCTAAGGGGATTCAGCA-3′ R: 5′-TGCAGGGTCCGAAAGTTTTG-3′
10	*Adamts5*	50	183	F: 5′-TCCTAGAAACAATGGCCGGT-3′ R: 5′-ACTCCAGCATACTTGGGGAC-3′
11	*Adamts9*	52	152	F: 5′-ATTTGGATCCTGGACACCGT-3′ R: 5′-GTGTCACGGTGCACATTTCT-3′
12	*Osteopontin*	59.2	198	F: 5′-GCAGCAGACACAGAATGACC-3′ R: 5′-TCTGTGGGGAAGTCTGTGAC-3′
13	*Runx2*	61.1	211	F: 5′-CCCCGGCTCCTCCCAAAACCAA-3′ R: 5′-CCGCCTCCACACCGTCACTCTG-3′
14	*Msx2*	53.8	162	F: 5′-TCCCTTTCCCCATCAACTCC-3′ R: 5′-TCACCGTGCCTTTCTGATCT-3′
15	*Osterix*	50.5	203	F: 5′-CTATAGGGGCGGTTCGGG-3′ R: 5′-CTCGGGTCGGTGTTATGGAT-3′
16	*β-Catenin*	56.1	205	F: 5′-ACTGTTCTACGCCATCACCA-3′ R: 5′-ACCACTGGCCAGAATGATGA-3′
17	*Wnt3a*	58	211	F: 5′-GTGAGGACGTGGAGTTT-3′ R: 5′-GAAGCGCTGTCGTATTTG-3′
18	*Dkk1*	51.9	166	F: 5′-CCGGAGCAGAAGGTTGTTTC-3′ R: 5′-CTGGAAACTCAGCGCGTAC-3′
19	*Bmp2*	55	238	F: 5′-GTTTGTGGTGGAGGTGGTTC-3′ R: 5′-GTCCACATACAACGGATGCC-3′
20	*Smad1*	51	323	F: 5′-GCAGGGAGATGAAGAAGA-3′ R: 5′-GCTTGTAGTGGTAAGGATTG-3′
21	*Smad5*	51	218	F: 5′-GGAAACAAGGAGATGAAGAG-3′ R: 5′-CCAGACCCGACAGTAAA-3′
22	*Smad8*	50	254	F: 5′-ACACCAGGAGACACATAG-3′ R: 5′-CATCTTCGTCAGCTCATATAC-3′
23	*Notch1*	53.8	233	F: 5′-GACATCGATGAGTGCAACCC-3′ R: 5′-TCCAGGTTGATCTCGCAGTT-3′
24	*Sox9*	56.1	191	F: 5′-CCGTTTTCTCCTCCCCTGAT-3′ R: 5′-CTCTTGAGGTCGGGTGTTCT-3′
25	*Pit2*	56	234	F: 5′-GCAGCAGATACATCAACTC-3′ R: 5′-AGACGTGTCCTTCTCTTC-3′
26	*β-Actin*	55	139	F: 5′-AGTACCCCATTGAACACGGT-3′ R: 5′-ATACATGGCTGGGGTGTTGA-3′

**Table 2:** qRT-PCR results of comparison between uninduced control and OS-induced AVICs.

			OS-induced AVICs
Osteogenic marker genes	*Osteopontin*, *Runx2*, *ALP*, *Msx2*, *Osterix*		All are upregulated

Negative regulatory molecule of bone cell differentiation	*Asporin*		Downregulated

AVICs marker genes	*Fibroblast*	*Col1a1*	Downregulated
	*Fibroblast*	*Vimentin*	Downregulated
	*Myofibroblast*	*Sm22α*	Upregulated
	*Extracellular matrix molecule*	*Fibronectin*	Upregulated
	*Cell adhesion molecule*	*N-Cadherin*	Upregulated
	*Matrix metalloproteinase*	*Adamts5*	Upregulated
	*Matrix metalloproteinase*	*Adamts9*	Downregulated

Intracellular phosphate level regulatory gene	*Na-dependent Pi transporter*	*PiT2*	Upregulated

Signaling-specific markers genes	*Wnt signaling*	*β-Catenin*	Upregulated
		*Wnt3a*	Upregulated
		*Dkk1*	Downregulated

	*Other signaling-specific marker genes*	*Bmp2*	Upregulated
		*Smad 1*	Downregulated
		*Smad 5*	Upregulated
		*Smad 8*	Upregulated
		*Notch 1*	Upregulated
		*Sox 9*	Downregulated

**Table 3:** qRT-PCR results of comparison between OS-induced control and OS+rhAsp-treated AVICs.

		OS+rhAsp-conditioned AVICs
Negative regulatory molecule of bone cell differentiation	*Asporin*	Upregulated

Osteogenic marker genes	*Osteopontin*, *Runx2*	Downregulated

Wnt signaling-specific markers genes	*β-Catenin*	Downregulated
	*Wnt3a*	Downregulated
	*Dkk1*	Upregulated

**Table 4:** qRT-PCR results of comparison among OS-induced control, OS+rhWnt3a-, and OS+rhWnt3a+rhAsp-treated AVICs.

		OS+rhWnt3a-conditioned AVICs	OS+rhWnt3a+rhAsp-conditioned AVICs
Negative regulatory molecule of bone cell differentiation	*Asporin*	Downregulated	Upregulated

Osteogenic marker genes	*Osteopontin*, *Runx2*	Upregulated	Downregulated

Wnt signaling-specific markers genes	*β-Catenin*	Upregulated	Downregulated
	*Wnt3a*	Upregulated	Downregulated
	*Dkk1*	Downregulated	Upregulated

**Table 5:** qRT-PCR results of comparison among OS-induced control, OS+Xav939-, and OS+Xav939+rhAsp-treated AVICs.

		OS+Xav939-conditioned AVICs	OS+Xav939+rhAsp-conditioned AVICs
Negative regulatory molecule of bone cell differentiation	*Asporin*	Unchanged	Upregulated
Osteogenic marker genes	*Osteopontin*, *Runx2*	Unchanged	Downregulated
Wnt signaling-specific markers genes	*β-Catenin*	Downregulated	Downregulated
	*Wnt3a*	Downregulated	Downregulated
	*Dkk1*	Upregulated	Upregulated

## Data Availability

All the generated data used to support the findings of this study are either included within the article or within the supplementary information file.

## Supplementary Materials

Supplementary Materials Supplementary Figure 1: optimization of the annealing temperatures of all the primers used for reverse-transcriptase PCR assays by temperature gradient PCR. For gene expression analyses, at first, all the primer sets are designed through the IDT PrimerQuest® tool. Then to detect the annealing temperature of each primer set, we have performed temperature gradient reverse-transcriptase PCR experiments. Here in this figure, we show 60-50°C gradient reverse-transcriptase PCR data (A) including Myh7, Gata4, Vimentin, ALP, Osteopontin, Msx2, *β*-catenin, Wnt3a, Dkk1, Notch1, Sox9, Osterix, and Bmp2. Next, we show 60-48°C gradient reverse-transcriptase PCR data (B) including Asporin, Col1a1, Sm22*α*, N-cadherin, and *β*-actin. Next, we show 65-55°C gradient reverse-transcriptase PCR data (C) including Fibronectin and Runx2. Next, we show 52-50°C gradient reverse-transcriptase PCR data (D) including Adamts5, Adamts9, Smad1, Smad5, and Smad8. Lastly, we show 56-52°C gradient reverse-transcriptase PCR data (E) of PiT2. All the detected annealing temperatures which were used for further gene expression analyses are mentioned in the primer list (Table 1) along with the product size and forward and reverse primer sequences. Supplementary Figure 2: determination of primer efficiencies by quantitative real-time PCR. To calculate primer efficiencies, we have followed Qiagen guidelines and performed the “efficiency and slope” generation step for important primer sets (Asporin, *β*-catenin, and Fibronectin). Here, we have taken 1 *μ*g of cDNA template of control AVICs sample, and it was serially diluted with nuclease free water as follows: 1 : 1, 1 : 10, 1 : 100, 1 : 1000, and 1 : 10000. Then, we did the standard curve derivation through the quantitative real-time PCR run. After getting the threshold values (Cq), these were plotted in *y*-axis and the logs of cDNA dilutions were plotted in *x*-axis (as shown in the figure). Slope (*S*) was determined in MS Excel, and the primer efficiency was calculated by the following formula: Primer efficiency (%) = [10^(−1/S) − 1] × 100. In this figure, we show the generated standard curves of Asporin, *β*-catenin, and Fibronectin in A, B, and C, respectively. Also the calculated primer efficiencies of these primers are 95.68% for Asporin, 98.84% for *β*-catenin, and 99.21% for Fibronectin. Supplementary Figure 3: detection of Asporin mRNA expression in embryonic outflow tract cushion and in adult aortic cusp. To determine the basal-level mRNA expression of Asporin, we have analyzed the expression by reverse-transcriptase PCR data in the isolated adult aortic valve tissue compared to embryonic day 5 (E5) outflow (OFT) tract cushion tissue. We show increased gene expression (according the more intense band in the adult tissue compared to embryonic OFT tissue). Here, we have used *β*-actin as loading control. Supplementary Figure 4: quantitative gene expression analyses of other osteogenic inducing pathways involved in osteogenesis after OS induction in adult AVICs in culture. Here, we have analyzed additional gene expression analyses taking signaling-specific marker genes comparing between control and OS-induced AVICs. BMP/Smad signaling-specific markers have been analyzed in A (I–IV) and Notch/Sox9 signaling-specific markers have been analyzed in B (I, II). Here, BMP2 has increased by 0.07 fold (A, I), Smad1 has decreased by 0.15 fold (A, II), and Smad5 and Smad8 increased by 7.51 and 6.11 folds (A, III and IV) in OS-treated AVICs compared to untreated control cells, respectively. Likewise, Notch1 mRNA has increased by 1.62 fold (B, I) and Sox9 mRNA has decreased by 0.67 fold (B, II) in OS-treated compared to untreated AVICs in culture. Supplementary Figure 5: cell viability after AVICs treatment with human recombinant Asporin (rhAsp) protein in culture. Here, we have shown cell viability analyses by performing DAPI staining after the recombinant Asporin protein (rhAsp) treatment on AVICs. The cells were seeded after counting in hemocytometer (0.6^×^10^4 cells/ml; 2 ml of such cell suspension is added in each plate) during the culture. Then, we have applied dose-dependent concentrations from 100 to 200 ng/ml of rhAsp. Then the cells were stained with DAPI and we have counted DAPI-positive cells as viable cells using this formula: (Total viable cells/Total cells) × 100. We show (A–D) that the determined percentages of control AVICs, 100 ng/ml rhAsp-treated AVICs, 150 ng/ml rhAsp-treated AVICs, and 200 ng/ml rhAsp-treated AVICs, are 88, 81.21, 93.81, and 76.7, respectively. Therefore, it has indicated that there are more than 75% DAPI-positive distinct nuclei indicating viable AVICs after rhAsp treatment in culture. Also, statistical analyses have been showing insignificant changes (E) in cell viability (DAPI^+^ nuclei) of rhAsp-treated AVICs compared to untreated controls in culture, where *p* > 0.05 and *n* = 3.
