# Supplementary material for: Asporin Reduces Adult Aortic Valve Interstitial Cell Mineralization Induced by Osteogenic Media and Wnt Signaling Manipulation In Vitro
Source: Int J Cell Biol. 2020 Apr 10;2020:2045969. doi: 10.1155/2020/2045969 (PMC7171660; doi:10.1155/2020/2045969)
Supplement: Supplementary Materials — Supplementary Figure 1: optimization of the annealing temperatures of all the primers used for reverse-transcriptase PCR assays by temperature gradient PCR. For gene expression analyses, at first, all the primer sets are designed through the IDT PrimerQuest® tool. Then to detect the annealing temperature of each primer set, we have performed temperature gradient reverse-transcriptase PCR experiments. Here in this figure, we show 60-50°C gradient reverse-transcriptase PCR data (A) including Myh7, Gata4, Vimentin, ALP, Osteopontin, Msx2, β-catenin, Wnt3a, Dkk1, Notch1, Sox9, Osterix, and Bmp2. Next, we show 60-48°C gradient reverse-transcriptase PCR data (B) including Asporin, Col1a1, Sm22α, N-cadherin, and β-actin. Next, we show 65-55°C gradient reverse-transcriptase PCR data (C) including Fibronectin and Runx2. Next, we show 52-50°C gradient reverse-transcriptase PCR data (D) including Adamts5, Adamts9, Smad1, Smad5, and Smad8. Lastly, we show 56-52°C gradient reverse-transcriptase PCR data (E) of PiT2. All the detected annealing temperatures which were used for further gene expression analyses are mentioned in the primer list (Table 1) along with the product size and forward and reverse primer sequences. Supplementary Figure 2: determination of primer efficiencies by quantitative real-time PCR. To calculate primer efficiencies, we have followed Qiagen guidelines and performed the “efficiency and slope” generation step for important primer sets (Asporin, β-catenin, and Fibronectin). Here, we have taken 1 μg of cDNA template of control AVICs sample, and it was serially diluted with nuclease free water as follows: 1 : 1, 1 : 10, 1 : 100, 1 : 1000, and 1 : 10000. Then, we did the standard curve derivation through the quantitative real-time PCR run. After getting the threshold values (Cq), these were plotted in y-axis and the logs of cDNA dilutions were plotted in x-axis (as shown in the figure). Slope (S) was determined in MS Excel, and the primer efficiency was calcul [file 2045969.f1.pdf]

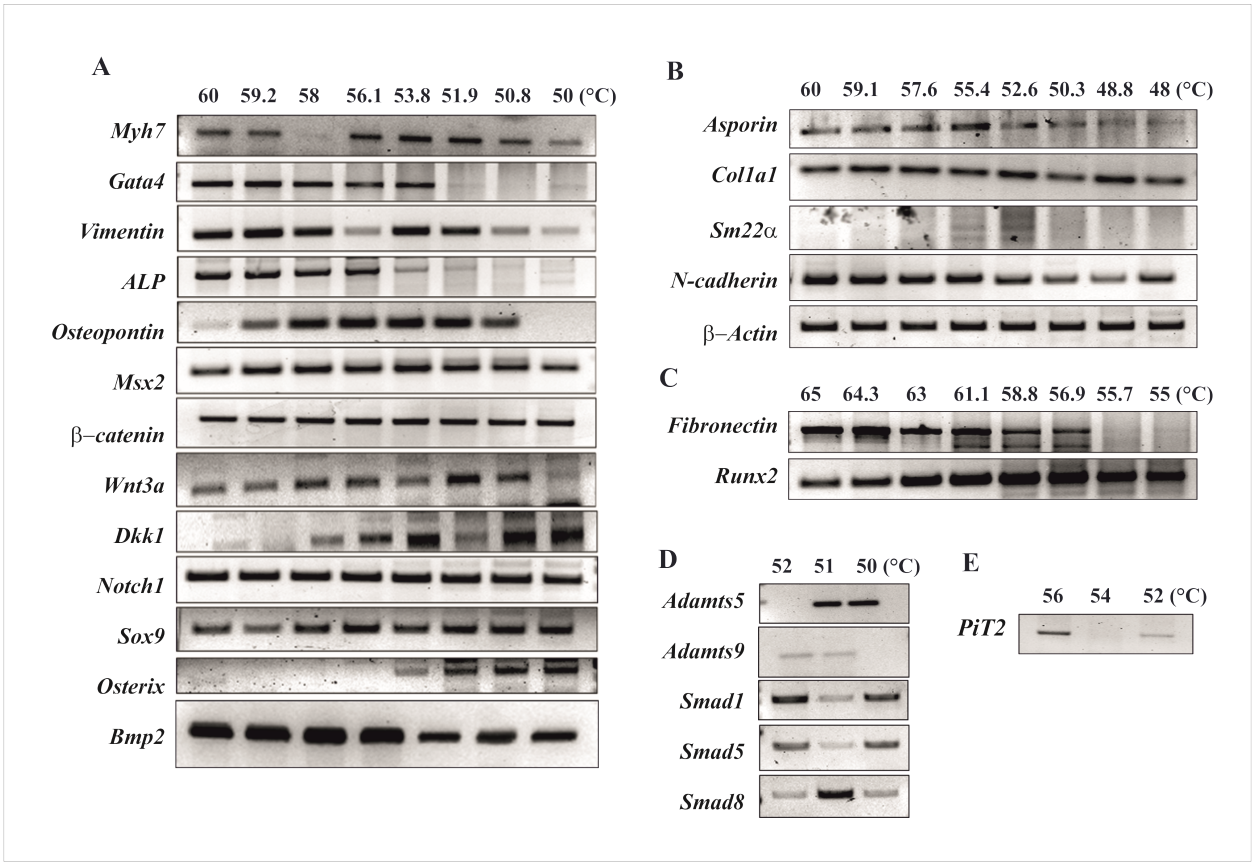

Supplementary figure I: **Optimization of the annealing temperatures of all the primers used for Reverse Transcriptase PCR assays by temperature gradient PCR. A:** 60°-50°C temperature gradient has been used for the *Myh7, Gata4, Vimentin, ALP, Osteopontin, Msx2, β-catenin, Wnt3a, Dkk1, Notch1, Sox9, Osterix* and *Bmp2*. **B:** 60°-48°C temperature gradient has been used for the *Asporin, Col1a1, Sm22α, N-cadherin* and *β-actin*. **C:** 65°-55°C temperature gradient has been used for the *Fibronectin* and *Runx2*. **D:** 52°-50°C temperature gradient has been used for the *Adamts5, Adamts9, Smad1, Smad5* and *Smad8*. **E:** 52°-56°C temperature

gradient has been used for the *PiT2*. Specific annealing temperature and product size for each set of primer is provided in Table 1.

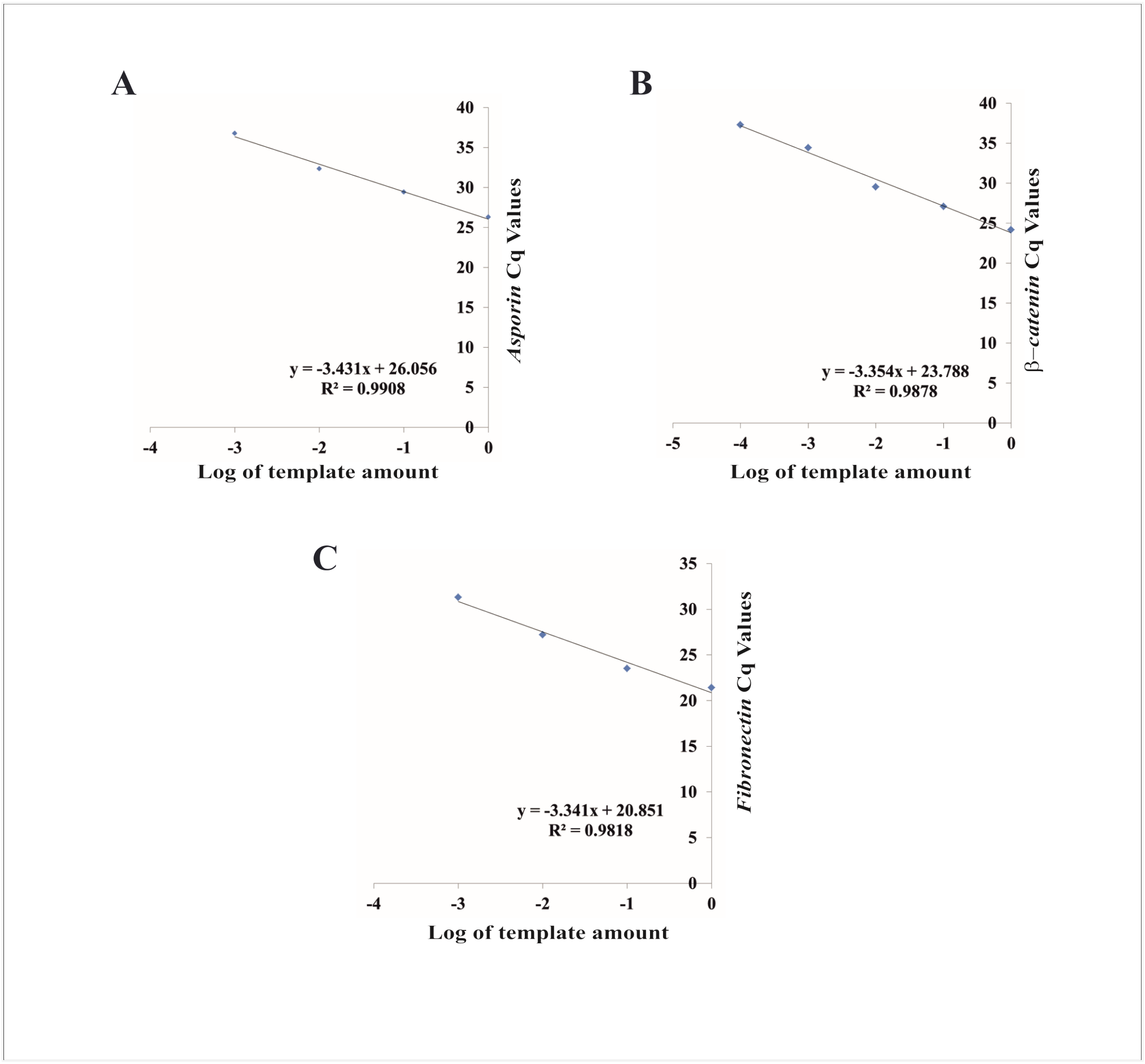

Supplementary figure II: **Derivation of the standard curves of the gene primers by quantitative Real Time PCR (A-C).** To determine the primer efficiencies threshold values (Cq) have been generated using template cDNA with serial dilution. Log of template cDNA has been plotted in X-axis and Cq values are plotted in Y-axis. **A:** Standard Curve of the *Asporin* gene primer (Calculated % efficiency = 95.68). **B:** Standard Curve of the *β-catenin* gene primer

(Calculated % efficiency = 98.84). **C:** Standard Curve of the *Fibronectin* gene primer (Calculated % efficiency = 99.21).

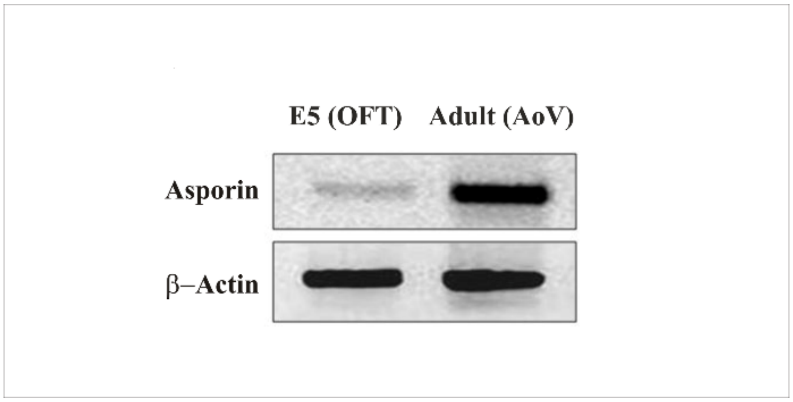

Supplementary figure III: **Detection of *Asporin* mRNA expression in embryonic outflow tract cushion and in adult aortic cusp.** Gene expression analysis by RT-PCR shows increased level of Asporin mRNA in isolated adult aortic valve tissue compared to embryonic day 5 (E5) outflow (OFT) tract cushion as detected by agarose gel band intensity. *β-actin* was used as loading control. (n=3)

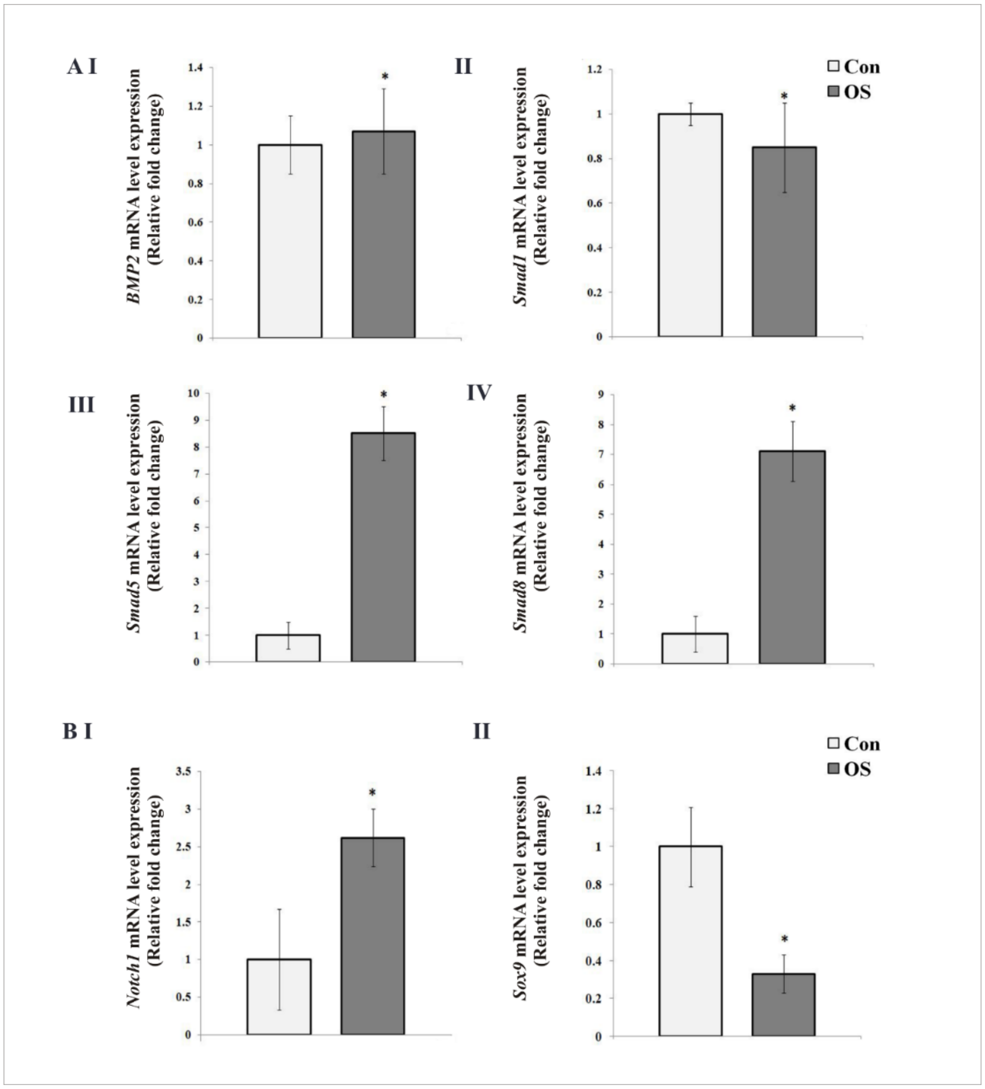

Supplementary figure IV: **Quantitative gene expression analyses of other osteogenic inducing pathways involved in osteogenesis after OS induction in adult AVICs in culture.** qRT-PCR data show that BMP/Smad signaling specific markers, *BMP2* is increased by 0.07 fold, *Smad1* is decreased by 0.15 fold, *Smad5* and *Smad8* are increased by 7.51 and 6.11 folds, respectively in OS treated AVICs compared to uninduced control AVICs. Expression of *Notch1* mRNA is increased by 1.62 fold and *Sox9* mRNA is decreased by 0.67 fold in OS treated AVICs compared to uninduced control AVICs. Statistical significance was determined by Student's *t*-test, where * denotes $p \leq 0.05$ and n=3.

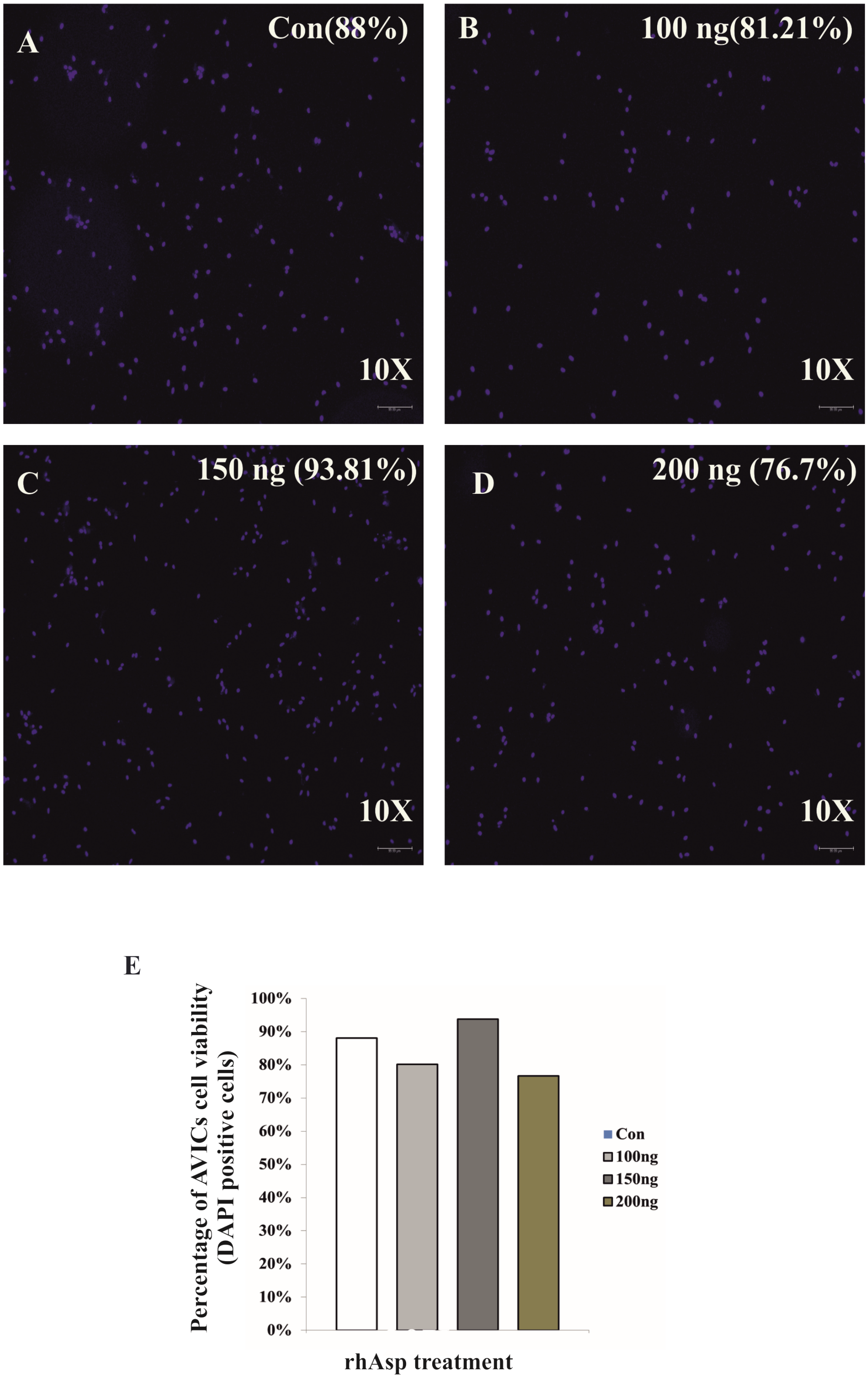
A
Con(88%)
10X
B
100 ng(81.21%)
10X
C
150 ng (93.81%)
10X
D
200 ng (76.7%)
10X
E
Percentage of AVICs cell viability (DAPI positive cells)
100%
90%
80%
70%
60%
50%
40%
30%
20%
10%
0%
Con
100ng
150ng
200ng
rhAsp treatment

Supplementary figure V: **Cell viability after AVICs treatment with human recombinant Asporin (rhAsp) in culture:** Dose dependent concentrations (100ng/ml, 150ng/ml and 200ng/ml) of rhAsp were applied to normal untreated AVICs to check the viability of cells. To mark viable cells, nuclei were stained with DAPI (blue). **A-D**: Microscopic images show that in all the conditions [control (A) 88%; in 100 ng/ml (B) 81.21%; in 150 ng/ml (C) 93.81%; and in 200 ng/ml (D) 76.7%], there are more than 75% DAPI positive distinct nuclei indicating viable AVICs after rhAsp treatment in culture. **E**: Statistical data also show insignificant changes in cell viability ($DAPI^+$ nuclei) of rhAsp treated AVICs compared to untreated controls in culture, where $p > 0.05$ and $n = 3$.
